# RFX1 downregulation contributes to TLR4 overexpression in CD14^+^ monocytes via epigenetic mechanisms in coronary artery disease

**DOI:** 10.1186/s13148-019-0646-9

**Published:** 2019-03-11

**Authors:** Pei Du, Keqin Gao, Yu Cao, Shuang Yang, Yang Wang, Ren Guo, Ming Zhao, Sujie Jia

**Affiliations:** 1 Department of Pharmacy, The Third Xiangya Hospital, Central South University, Changsha, China; 2 Department of Cardiology, The Third Xiangya Hospital, Central South University, Changsha, China; 3 Department of Dermatology, The Second Xiangya Hospital, Central South University, Hunan Key Laboratory of Medical Epigenomics, Changsha, China; 4 Center of Clinical Pharmacology, The Third Xiangya Hospital, Central South University, Changsha, China; 5 Department of Pharmacy, Weifang People’s Hospital, Weifang, China

**Keywords:** Coronary artery diseases, CD14^+^ monocytes, Epigenetic, Toll-like receptor 4, Regulatory factor X1

## Abstract

**Background:**

Toll-like receptor 4 (TLR4) expression is increased in activated monocytes, which play a critical role in the pathogenesis of coronary artery disease (CAD). However, the mechanism remains unclear. Regulatory factor X1 (RFX1) is a critical transcription factor regulating epigenetic modifications. In this study, we investigated whether RFX1 and epigenetic modifications mediated by RFX1 contributed to the overexpression of TLR4 in activated monocytes.

**Results:**

Compared with those of the controls, the mRNA and protein expression of RFX1 were downregulated and the mRNA expression of TLR4 was upregulated in CD14^+^ monocytes obtained from CAD patients and CD14^+^ monocytes obtained from healthy controls treated with low-density lipoprotein (LDL). The mRNA expression of RFX1 was negatively correlated with the mRNA expression of TLR4 in CD14^+^ monocytes. RFX1 knockdown led to the overexpression of TLR4 and the activation of CD14^+^ monocytes. In contrast, the overexpression of RFX1 inhibited TLR4 expression and the activation of CD14^+^ monocytes stimulated with LDL. Moreover, *TLR4* was identified as a target gene of RFX1. The results indicated that RFX1 downregulation contributed to the decreased DNA methylation and histone H3 lysine 9 trimethylation and the increased H3 and H4 acetylation in the *TLR4* promoter via the lack of recruitments of DNA methyltransferase 1 (DNMT1), histone deacetylase 1 (HDAC1), and histone-lysine *N*-methyltransferase SUV39H1 (SUV39H1), which were observed in CD14^+^ monocytes of CAD patients.

**Conclusions:**

Our results show that RFX1 expression deficiency leads to the overexpression of TLR4 and the activation of CD14^+^ monocytes in CAD patients by regulating DNA methylation and histone modifications, which highlights the vital role of RFX1 in the pathogenesis of CAD.

**Electronic supplementary material:**

The online version of this article (10.1186/s13148-019-0646-9) contains supplementary material, which is available to authorized users.

## Background

Cardiovascular disease (CVD) is the predominant cause of mortality worldwide. The leading cause of death in CVD is atherosclerosis (AS) and its thrombotic complications. In the coronary artery, AS contributes to coronary artery disease (CAD), which includes stable angina pectoris and acute coronary syndrome [1]. Monocytes, components of the innate immune system, play an essential role in the initiation and development of AS [2, 3]. Studies have indicated the mRNA and protein expression of Toll-like receptor 4 (TLR4) was significantly increased in circulating monocytes from AS and CAD patients [4, 5]. In ApoE knockout mice, which develops atherosclerotic plaques with morphology resembling human AS [6], a significant increase in TLR4 expression was identified via flow cytometric analyses in monocytes [7]. Furthermore, TLR4 expression and the activation of the downstream signaling pathway initiated the hyperactivation of monocytes [8]. Cytokines downstream of the TLR4 signaling pathway, such as IL-6, IL-1β, MCP-1, and TNF-α, were also overproduced in monocytes from CAD patients, particularly acute coronary syndrome (ACS) patients [9–12]. Moreover, the adhesion and migration abilities of monocytes were significantly increased by increasing the expression of adhesion molecules and chemokine receptors in activated monocytes from AS [13, 14]. Low-density lipoprotein (LDL) is one of the risk factors of AS. Monocytes exposed to oxidized low density lipoprotein (ox-LDL) increased the expression of TLR4 and the production of pro-inflammatory cytokines such as TNF-α, IL-6, MCP-1, IL-18, and CD36, thus improving the adhesion and migration abilities of monocytes [15–17].

Recent studies have indicated that epigenetic regulation plays a critical role in the pathogenesis of CAD. A previous study has shown global deoxyribonucleic acid (DNA) hypermethylation in the blood samples of patients with CAD [18]. The DNA methylation levels of the long interspersed element-1 (LINE-1) and ATP-binding cassette A1 (ABCA1) promoters were significantly increased as CAD risks increased [19, 20]. In the macrophages of ox-LDL-treated apoE^−/−^ mice, the upregulation of DNMTs was associated with DNA hypermethylation in the cystathionine-gamma-lyase (CSE) gene promoter, which resulted in the inflammation of macrophages and the acceleration of atherosclerosis [20]. Many studies have demonstrated that HDACs were involved in regulating aberrant gene transcription in CAD [21–23]. HDAC activity was likely to play a significant role in inducing severe myocardial ischemia and reperfusion damage [24, 25]. A recent study indicated that aberrant histone acetylation and trimethylation were identified in the *MCP*-*1* gene promoter in monocytes from CAD patients [26]. However, the mechanism of aberrant epigenetic modifications in CAD patients remains unclear.

Recent evidence has shown that transcription factors, such as regulatory factor X1 (RFX1), are involved in regulating epigenetic modifications [27, 28]. RFX1 belongs to the regulatory factor protein of the X-box (RFX) family, which is characterized by a highly conserved 76-amino-acid DNA-binding domain and includes seven members (RFX1–7) [29]. RFX1 is the first cloned member of the RFX family, and has both a C-terminal repressive region overlapping a dimerization domain and an N-terminal activation domain [30]. It has been reported that the expression of RFX1 was decreased in the CD4^+^ T cells of lupus patients [31]. RFX1 mediated dimerization and transcriptional repression functions by recruiting epigenetic enzymes, such as DNA methyltransferase 1 (DNMT1), histone deacetylase 1 (HDAC1), and histone-lysine *N*-methyltransferase SUV39H1 (SUV39H1) [32]. RFX1 downregulation caused CD11a, CD70, and IL17A overexpression by reducing DNA methylation and increasing H3 acetylation levels in the promoter region of *CD11a*, *CD70*, and *IL17A* in the CD4^+^ T cells of systemic lupus erythematosus (SLE) patients, which contributed to autoimmune responses [31, 33].

In this study, we found that RFX1 expression was downregulated in CD14^+^ monocytes of CAD patients, which led to the overexpression of the target gene *TLR4* through the recruitment of DNMT1, HDAC1, and SUV39H1 to regulate DNA methylation and histone modifications in the *TLR4* promoter. These findings demonstrate the role and mechanism of RFX1 in regulating epigenetic modifications and the activation of monocytes in CAD patients, which suggests a novel therapeutic target for CAD.

## Results

### RFX1 and TLR4 expression changes in CD14^+^ monocytes from CAD patients and healthy subjects treated with LDL

**Fig. 1:**
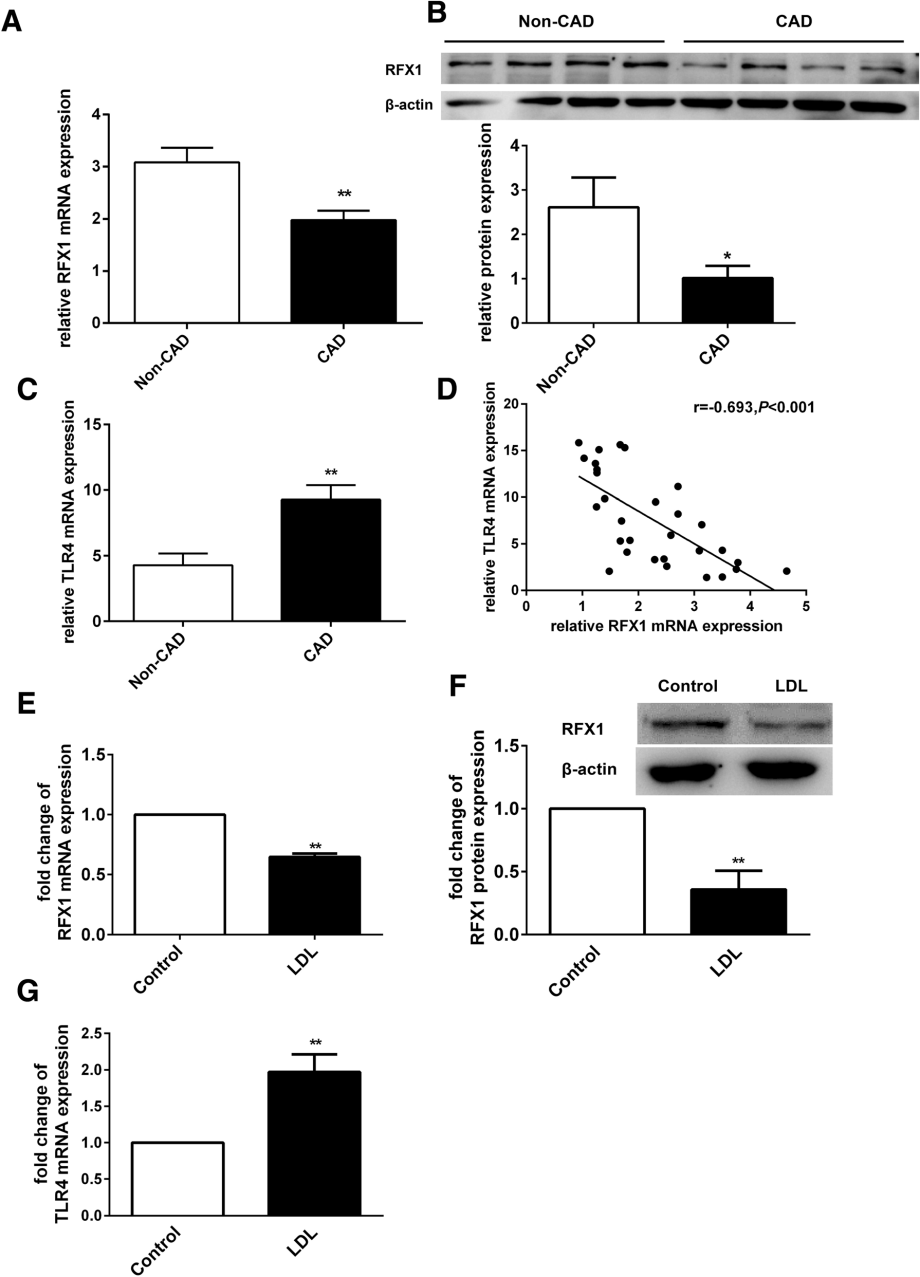
RFX1 and TLR4 expression changes in CD14^+^ monocytes from CAD patients and in the LDL-treated CD14^+^ monocytes. **a** The RFX1 mRNA expression level was determined by RT-qPCR in CD14^+^ monocytes from CAD patients (*n* = 16) and from non-CAD controls (*n* = 14). **b** The RFX1 protein expression level was determined by Western blotting in CD14^+^ monocytes from CAD patients (*n* = 10) and non-CAD controls (*n* = 10). **c** The TLR4 mRNA expression level was determined by RT-qPCR in CD14^+^ monocytes from CAD patients (*n* = 16) and from non-CAD controls (*n* = 14). **d** The correlation analysis between RFX1 mRNA levels and TLR4 mRNA levels. **e** The fold change of RFX1 mRNA expression was determined by RT-qPCR in the LDL-treated CD14^+^ monocytes compared with negative controls (/Control). **f** The fold change of RFX1 protein expression was determined by Western blotting in the LDL-treated CD14^+^ monocytes compared with negative controls (/Control). **g** The fold change of TLR4 mRNA expression was determined by RT-qPCR in the LDL-treated CD14^+^ monocytes compared with negative controls (/control). The values are the average of at least three biological replicates in **e**, **f**, and all data shown are the means ± SDs. *P < 0.05; **P < 0.01 relative to control

We detected the mRNA and protein expression levels of RFX1 in CD14^+^ monocytes from CAD patients and non-CAD controls. As shown in Fig. 1a, the real-time quantitative polymerase chain reaction (RT-qPCR) analysis indicated that the expression level of RFX1 mRNA was downregulated in CD14^+^ monocytes from the CAD patients (*n* = 16) compared with those from the non-CAD controls (*n* = 14). Moreover, we found that the protein expression level of RFX1 was decreased in CD14^+^ monocytes from the CAD patients (*n* = 10) compared with those from the non-CAD controls (*n* = 10) (Fig. 1b). Studies have indicated the mRNA and protein expression of TLR4 was significantly increased in circulating monocytes from AS and CAD patients [4, 5]. Here, we found the mRNA expression of TLR4 was increased in CD14^+^ monocytes from the CAD patients (*n* = 16) compared with those from the non-CAD controls (*n* = 14) (Fig. 1c), which was negatively correlated with the mRNA expression of RFX1 (Fig. 1d). The sequences of the primers are shown in Additional file 1: Table S1.

We subsequently investigated the effect of LDL on the expression of RFX1 in CD14^+^ monocytes. We isolated CD14^+^ monocytes from healthy subjects, treated the monocytes with LDL, and detected the mRNA and protein expression of RFX1 and TLR4 mRNA expression after LDL treatment. The results showed that the mRNA and protein expression of RFX1 were downregulated in the CD14^+^ monocytes after LDL treatment compared with the negative controls (Fig. 1e, f), while the mRNA expression of TLR4 was upregulated in the CD14^+^ monocytes with LDL treatment compared with the negative controls (Fig. 1g).

### RFX1 knockdown promotes TLR4 expression and the activation of CD14^+^ monocytes

**Fig. 2:**
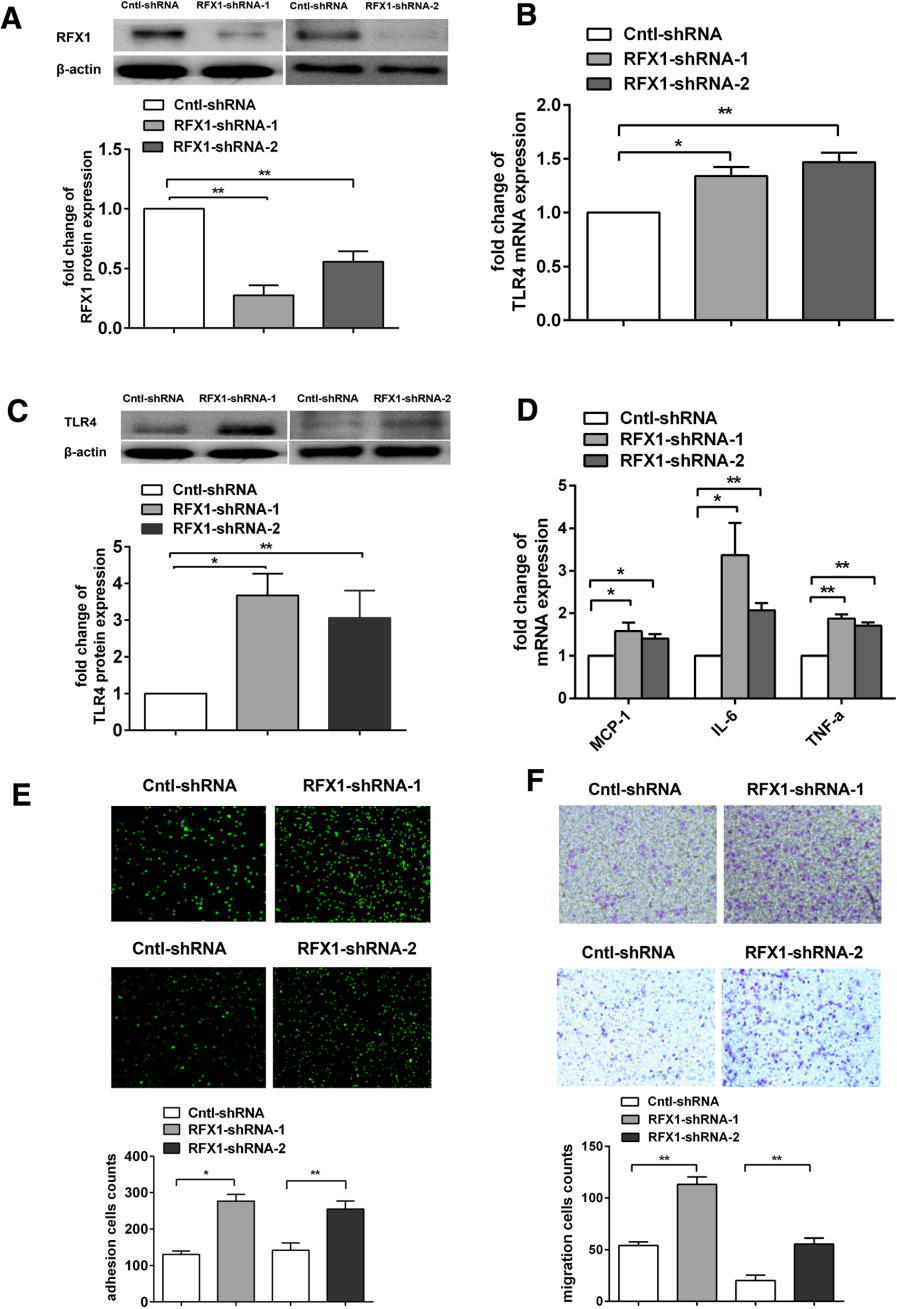
Knockdown of RFX1 increased TLR4 expression and promoted CD14^+^ monocyte activation. **a** The fold change of RFX1 protein expression was determined by western blotting in CD14^+^ monocytes transfected with RFX1-shRNA-1 or RFX1-shRNA-2 compared with Cntl-shRNA (/Cntl-shRNA). **b**, **c** The fold change of mRNA expression (**b**) and protein (**c**) of TLR4 were determined by RT-qPCR and western blotting in CD14^+^ monocytes transfected with RFX1-shRNA-1 or RFX1-shRNA-2 compared with Cntl-shRNA (/Cntl-shRNA). **d** The fold change of mRNA expression of TNF-α, IL-6 and MCP-1 were determined by RT-qPCR in CD14^+^ monocytes transfected with RFX1-shRNA-1 or RFX1-shRNA-2 compared with Cntl-shRNA (/Cntl-shRNA). **e** The adhesive capacity of CD14^+^ monocytes transfected with RFX1-shRNA-1, RFX1-shRNA-2, or with Cntl-shRNA was assessed using a fluorescence microscope (× 100). **f** The migration capacity of CD14^+^ monocytes transfected with RFX1-shRNA-1, RFX1-shRNA-2, or with Cntl-shRNA was assessed by a Transwell migration assay with a fluorescence microscope (× 100). All values are the average of at least three biological replicates, and the data shown are the means ± SDs. *P < 0.05; **P < 0.01 relative to control

We investigated whether RFX1 is involved in regulating TLR4 expression in monocytes, which has been confirmed to be critical for the activation of monocytes [34]; we knocked down RFX1 expression in CD14^+^ monocytes from healthy subjects by transfection with two RFX1-shRNA lentiviral vectors (RFX1-shRNA-1 and RFX1-shRNA-2) respectively. Compared with the monocytes transfected with the negative control (Cntl-shRNA), the CD14^+^ monocytes demonstrated decreased RFX1 protein expression at 72 h after RFX1-shRNA-1 and RFX1-shRNA-2 transfection (Fig. 2a). Moreover, both the mRNA and protein expression of TLR4 in the CD14^+^ monocytes transfected with RFX1-shRNA-1 and RFX1-shRNA-2 were markedly increased compared with those in the negative controls (Fig. 2b, c). In addition, the mRNA expression of TNF-α, IL-6, and MCP-1 (Fig. 2d) were significantly upregulated in the CD14^+^ monocytes transfected with RFX1-shRNA-1 and RFX1-shRNA-2 compared with the negative controls.

Previous studies have shown that the TLR4 expression level is relevant to the adhesive and chemotactic abilities of monocytes [35, 36]. Therefore, we also detected the adhesive and chemotactic capacities of CD14^+^ monocytes with RFX1 knockdown. The cell counting results showed that the number of adherent monocytes was significantly increased in the CD14^+^ monocytes transfected with RFX1-shRNA-1 and RFX1-shRNA-2 compared with the negative controls (Fig. 2e). The migration ability of the monocytes was assessed by the transwell migration assay, and the migration capacity of the CD14^+^ monocytes was assessed by the number of monocytes that migrated from the upper chamber to the lower chamber. The cell counting results showed that the number of migrated monocytes was significantly increased in the CD14^+^ monocytes transfected with RFX1-shRNA-1 and RFX1-shRNA-2 compared with the negative controls (Fig. 2f).

### RFX1 overexpression inhibits TLR4 expression and the activation of CD14^+^ monocytes induced by LDL

**Fig. 3:**
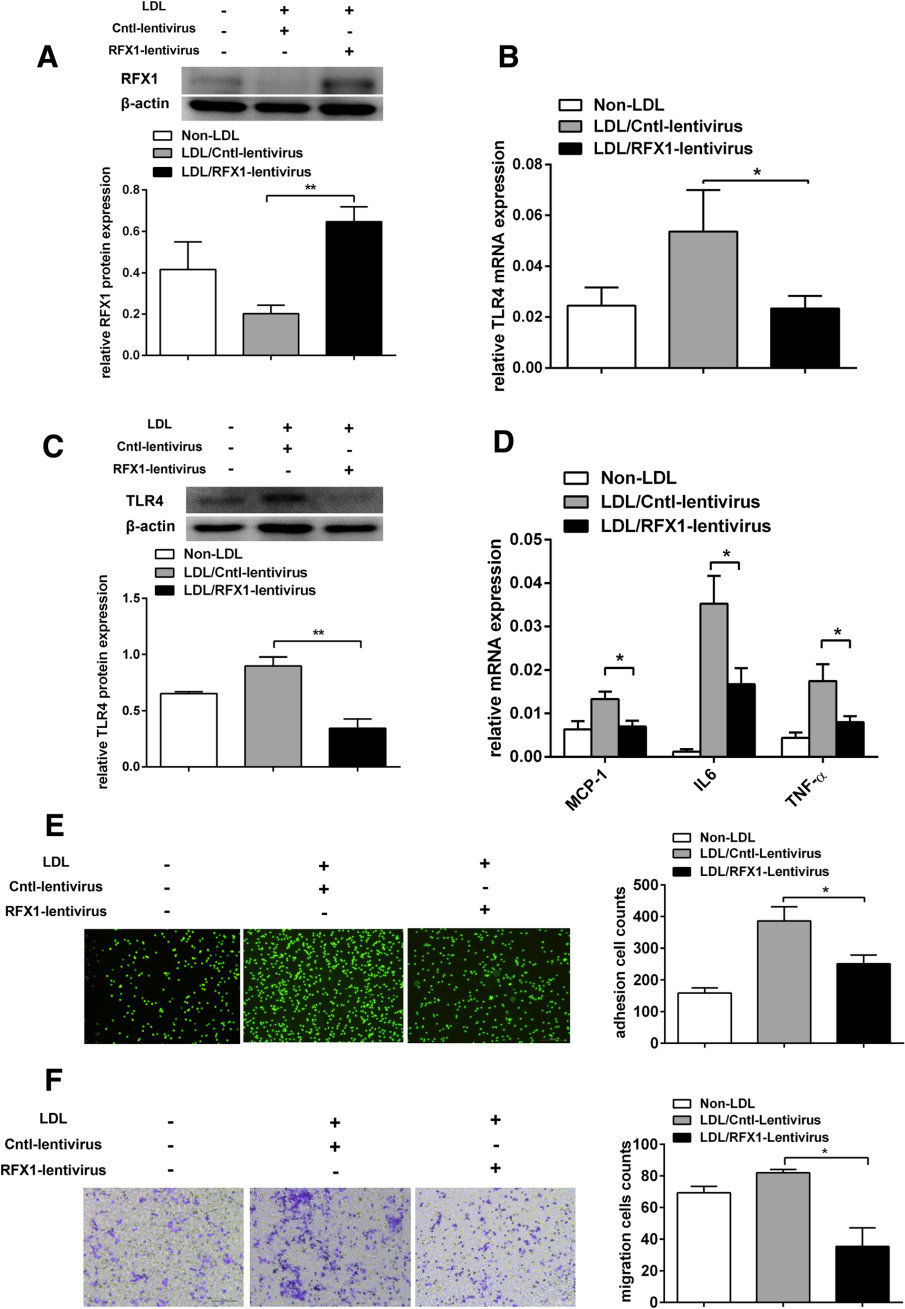
Overexpression of RFX1 decreased TLR4 expression and inhibited CD14^+^ monocyte activation induced by LDL. **a** The relative RFX1 protein expression was determined by Western blotting in LDL treated CD14^+^ monocytes transfected with RFX1-lentivirus or with Cntl-lentivirus and non-LDL-treated CD14^+^ monocytes. **b**, **c** The relative TLR4 mRNA (**b**) and protein (**c**) expression were determined by RT-qPCR and western blotting in three groups. **d** The relative mRNA expression of TNF-α, IL-6, and MCP-1 were determined by RT-qPCR in three groups. **e** The adhesive capacity of CD14^+^ monocytes in three groups was assessed using a fluorescence microscope (× 100). **f** The migration capacity of CD14^+^ monocytes in three groups was assessed by a Transwell migration assay with a fluorescence microscope (× 100). All values are the average of at least 3 biological replicates, and the data shown are the means ± SDs. *P < 0.05; **P < 0.01 relative to control

Furthermore, we investigated whether upregulating RFX1 can reverse the overexpression of TLR4 and the activation of CD14^+^ monocytes induced by LDL. We transfected LDL-treated CD14^+^ monocytes with an RFX1 lentiviral expression vector (RFX1-lentivirus) or empty lentivirus (Cntl-lentivirus). Compared with the monocytes transfected with empty lentivirus, LDL-treated CD14^+^ monocytes exhibited a significantly increased RFX1 protein expression at 72 h after RFX1-lentivirus transfection (Fig. 3a). Moreover, both the mRNA and protein expression of TLR4 in the LDL-treated CD14^+^ monocytes transfected with RFX1-lentivirus were markedly decreased compared with those in the empty controls (Fig. 3b, c). Moreover, compared with the empty controls, the LDL-treated CD14^+^ monocytes transfected with RFX1-lentivirus exhibited downregulated mRNA expression of TNF-α, IL-6, and MCP-1 and decreased adhesive and migration abilities (Fig. 3d–f).

### *TLR4* is a target gene of RFX1

**Fig. 4:**
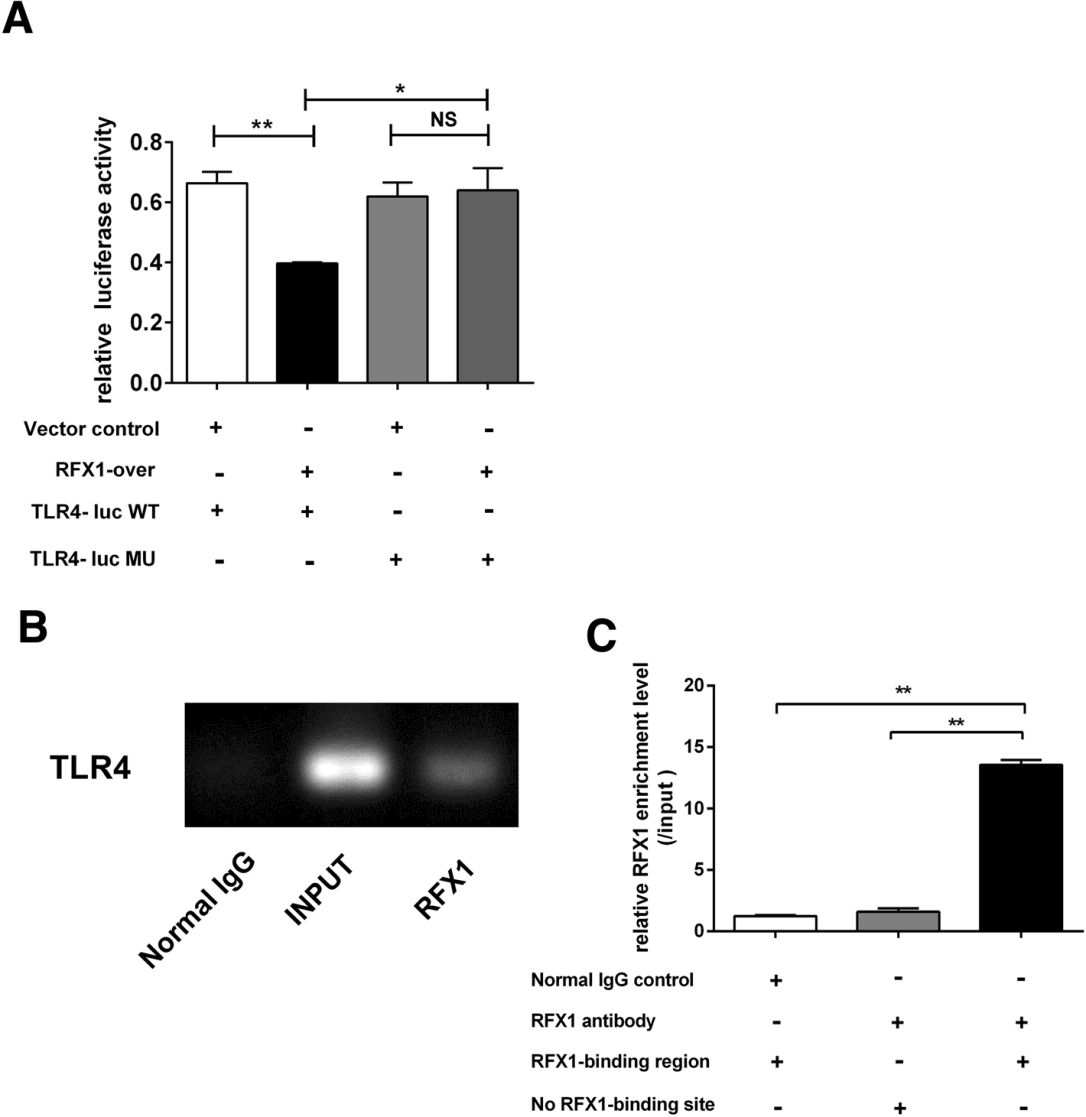
*TLR4* is a target gene of RFX1. **a** Relative luciferase activities in HEK293T cells cotransfected with RFX1 or with empty vector (negative control) and TLR4-luciferase reporter vectors. **b** Gel electrophoresis of ChIP-PCR product indicated the binding of RFX1 to the *TLR4* promoter in CD14^+^ monocyte cells. **c** ChIP-qPCR indicated the binding of RFX1 to the *TLR4* promoter in CD14^+^ monocytes. All values are the average of at least three biological replicates, and the data shown are the means ± SDs. *P < 0.05; **P < 0.01 relative to control

We performed luciferase reporter and ChIP-qPCR assays to investigate whether RFX1 could bind to the putative binding sites in the promoter of *TLR4*. DNA fragment that contained the wild-type RFX1 binding sites or mutant RFX1 binding sites in the promoter regions of *TLR4* were cloned into pGL3 vectors upstream of luciferase reporter gene (TLR4-luc WT and TLR4-luc MU). Two types of recombinant plasmids were separately cotransfected into HEK293T cells together with empty vectors (blank control) or plasmid-encoded RFX1 cDNA. As shown in Fig. 4a, the overexpression of RFX1 decreased the luciferase activity in the TLR4-luc-WT group compared with the blank control group; however, the mutation of the RFX1 binding sites in the *TLR4* promoter abrogated the repressive effects of RFX1 overexpression on the TLR4-luciferase activity in the HEK293T cells. Results of ChIP-PCR gel electrophoresis and ChIP-qPCR confirmed that RFX1 binds to the DNA fragment of the *TLR4* promoter region in CD14^+^ monocytes (Fig. 4b, c). Therefore, these data indicate that *TLR4* is a target gene of RFX1.

### RFX1 regulates the epigenetic modifications of the *TLR4* promoter in CD14^+^ monocytes

**Fig. 5:**
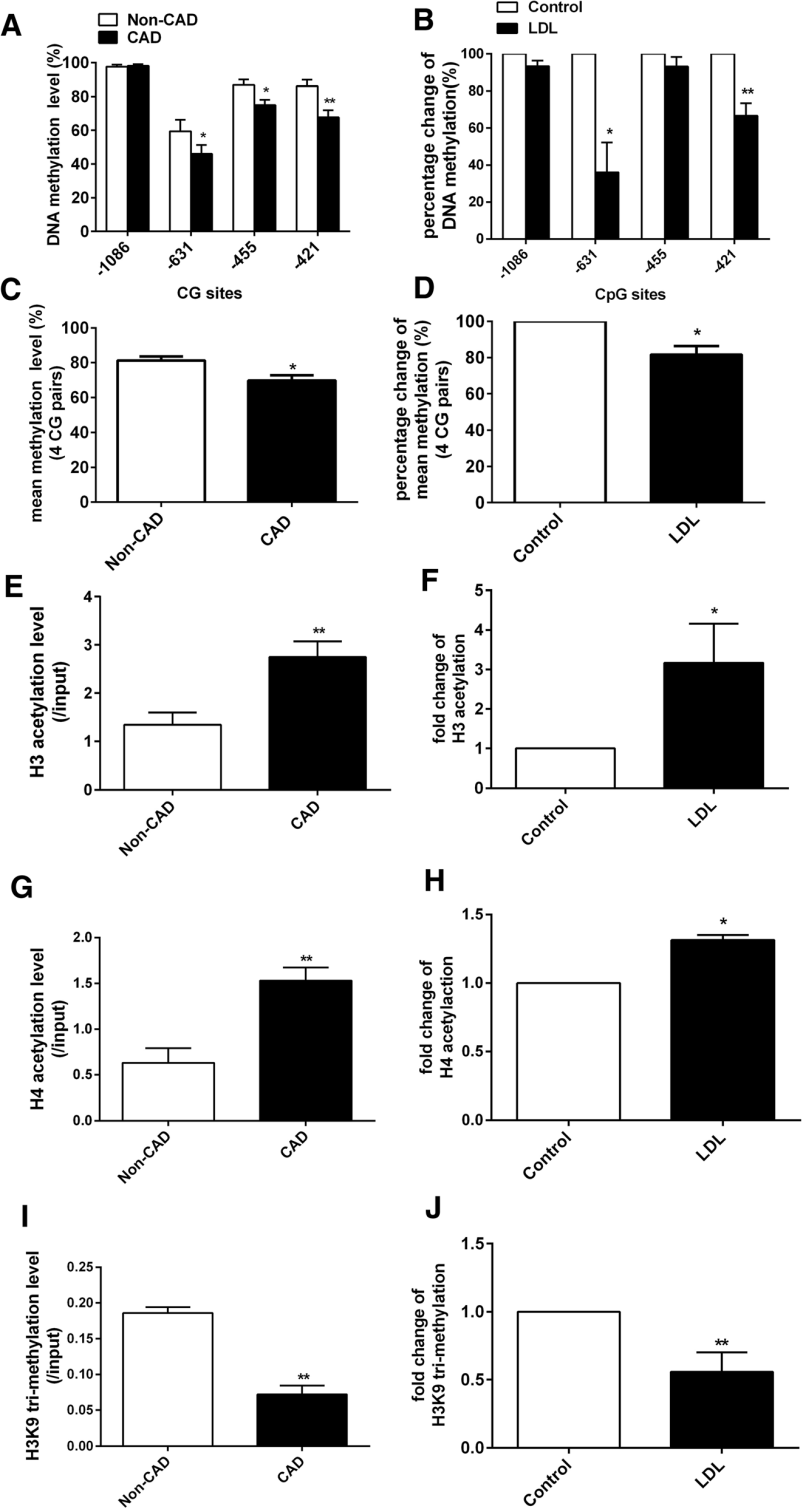
Epigenetic modifications status in the *TLR4* promoter in CAD patients and LDL-treated CD14^+^ monocytes. **a** The DNA methylation levels of 4 CG pairs (from − 1086 bp to − 421 bp upstream of the *TLR4* TSS) was detected by bisulfite sequencing in CAD CD14^+^ monocytes. **b** DNA methylation levels of 4 CG pairs (from − 1086 bp to − 421 bp upstream of the *TLR4* TSS) was detected by bisulfite sequencing. The percentage change of methylation level in each CG pair was shown between LDL-treatment and control group. **c** The mean DNA methylation level of all the 4 CG pairs (from − 1086 bp to − 421 bp upstream of the *TLR4* TSS) was calculated in CAD CD14^+^ monocytes. **d** The mean DNA methylation of all the 4 CG pairs (from − 1086 bp to − 421 bp upstream of the *TLR4* TSS) was calculated. The percentage change of mean methylation was shown between LDL treatment and control group. **e** The H3 acetylation level in *TLR4* promoter with RFX1 binding region were detected by ChIP-qPCR in CAD CD14^+^ monocytes. **f** The fold change of H3 acetylation in *TLR4* promoter with RFX1 binding region were detected by ChIP-qPCR in LDL-treated CD14^+^ monocytes. **g** The H4 acetylation level in *TLR4* promoter with RFX1 binding region were detected by ChIP-qPCR in CAD CD14^+^ monocytes. **h** The fold change of H4 acetylation in *TLR4* promoter with RFX1 binding region were detected by ChIP-qPCR in LDL-treated CD14^+^ monocytes. **i** The H3K9 trimethylation level in *TLR4* promoter with RFX1 binding region were detected by ChIP-qPCR in CAD CD14^+^ monocytes. **j** The fold change of H3K9 trimethylation in *TLR4* promoter with RFX1 binding region were detected by ChIP-qPCR in LDL-treated CD14^+^ monocytes. The fold changes were shown between LDL treatment and control group. All values are the average of at least three biological replicates, and the data shown are the means ± SDs. *P < 0.05; **P < 0.01 relative to control

Previous studies have indicated that the expression level of TLR4 was regulated by DNA methylation and histone acetylation [37, 38]. Here, we initially detected the epigenetic states of the *TLR4* promoter in CD14^+^ monocytes of CAD patients and in LDL-treated CD14^+^ monocytes. The bisulfite sequencing results showed that the methylation levels of the three CG sites − 631 bp, − 455 bp, and − 421 bp upstream of transcription start site (TSS) of *TLR4* genes were decreased in the CAD CD14^+^ monocytes compared with the non-CAD controls (Fig. 5a). The methylation of both − 631 bp and − 421 bp CG sites upstream of TSS of *TLR4* genes were also decreased in the LDL-treated CD14^+^ monocytes compared with the negative controls (Fig. 5b). The mean DNA methylation of all the four CG sites in the *TLR4* promoter was significantly reduced in both the CD14^+^ monocytes from CAD patients (Fig. 5c) and the LDL-treated CD14^+^ monocytes (Fig. 5d). ChIP-qPCR analysis indicated that the acetylation of H3 (Fig. 5e, f) and H4 (Fig. 5g, h) in RFX1 binding region of the *TLR4* promoter were significantly increased, while the trimethylation of H3K9 (Fig. 5i, j) in RFX1 binding region of the *TLR4* promoter was significantly decreased in the CD14^+^ monocytes from CAD patients and the LDL-treated CD14^+^ monocytes compared with the non-CAD controls or negative controls, respectively.

**Fig. 6:**
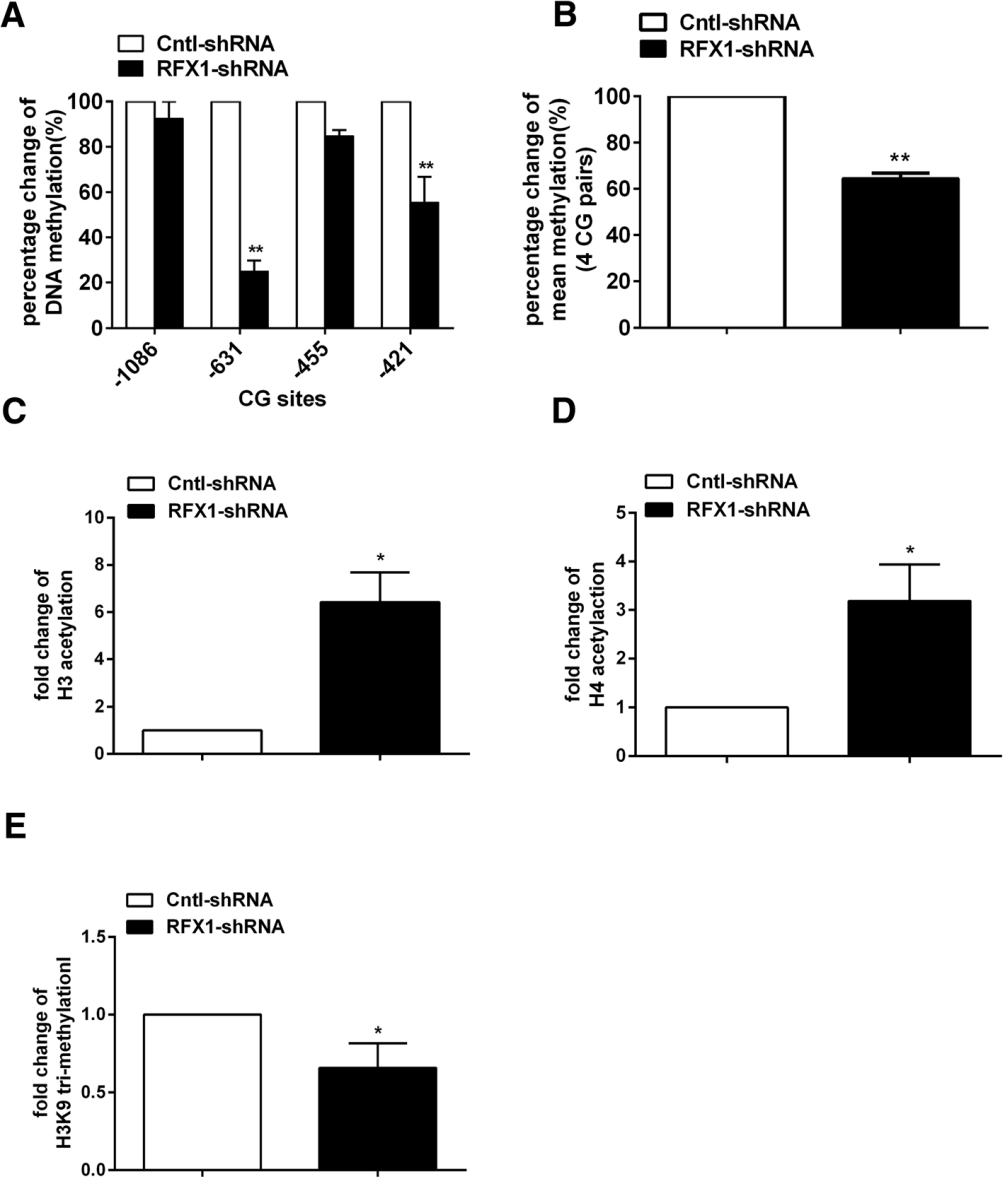
Epigenetic modifications of the *TLR4* promoter in CD14^+^ monocytes with RFX1 knockdown. **a** The percentage change of DNA methylation of four CG pairs (from − 1086 bp to − 421 bp upstream of the *TLR4* TSS) was detected by bisulfite sequencing in CD14^+^ monocytes transfected with RFX1-shRNA-1 compared with Cntl-shRNA (/Cntl-shRNA). **b** The percentage change of mean DNA methylation of all the four CG pairs (from − 1086 bp to − 421 bp upstream of the *TLR4* TSS) was detected by bisulfite sequencing in CD14^+^ monocytes transfected with RFX1-shRNA-1 compared with Cntl-shRNA (/Cntl-shRNA). **c**–**e** The fold changes of H3 (**c**), H4 (**d**) acetylation, and the H3K9 trimethylation in *TLR4* promoter with RFX1 binding region were detected by ChIP-qPCR in CD14^+^ monocytes transfected with RFX1-shRNA-1 compared with Cntl-shRNA. All values are the average of at least three biological replicates, and the data shown are the means ± SDs. *P < 0.05; **P < 0.01 relative to control

We subsequently investigated whether the aberrant expression and epigenetic modifications of TLR4 were regulated by RFX1 in the monocytes. First, we detected the epigenetic status of the *TLR4* promoter in monocytes with RFX1 knockdown. The bisulfite sequencing results showed that the methylation of two CG sites at positions − 631 bp and − 421 bp upstream of TSS of *TLR4* gene were significantly decreased and that the methylation of the CG site − 455 bp upstream of TSS of *TLR4* gene was nonsignificantly decreased in the CD14^+^ monocytes transfected with RFX1-shRNA-1 compared with the negative control monocytes (Fig. 6a). The mean DNA methylation of the four CG sites in the *TLR4* promoter was significantly decreased after RFX1-shRNA-1 transfection (Fig. 6b). ChIP-qPCR analysis indicated that the acetylation of H3 and H4 were significantly increased and the trimethylation of H3K9 was significantly decreased in the RFX1 binding region of *TLR4* promoter in the CD14^+^ monocytes with RFX1-shRNA-1 transfection compared with negative controls (Fig. 6c–e). However, no significant change was observed in the region without RFX1 binding site (Additional file 2: Figure S1A-C).

**Fig. 7:**
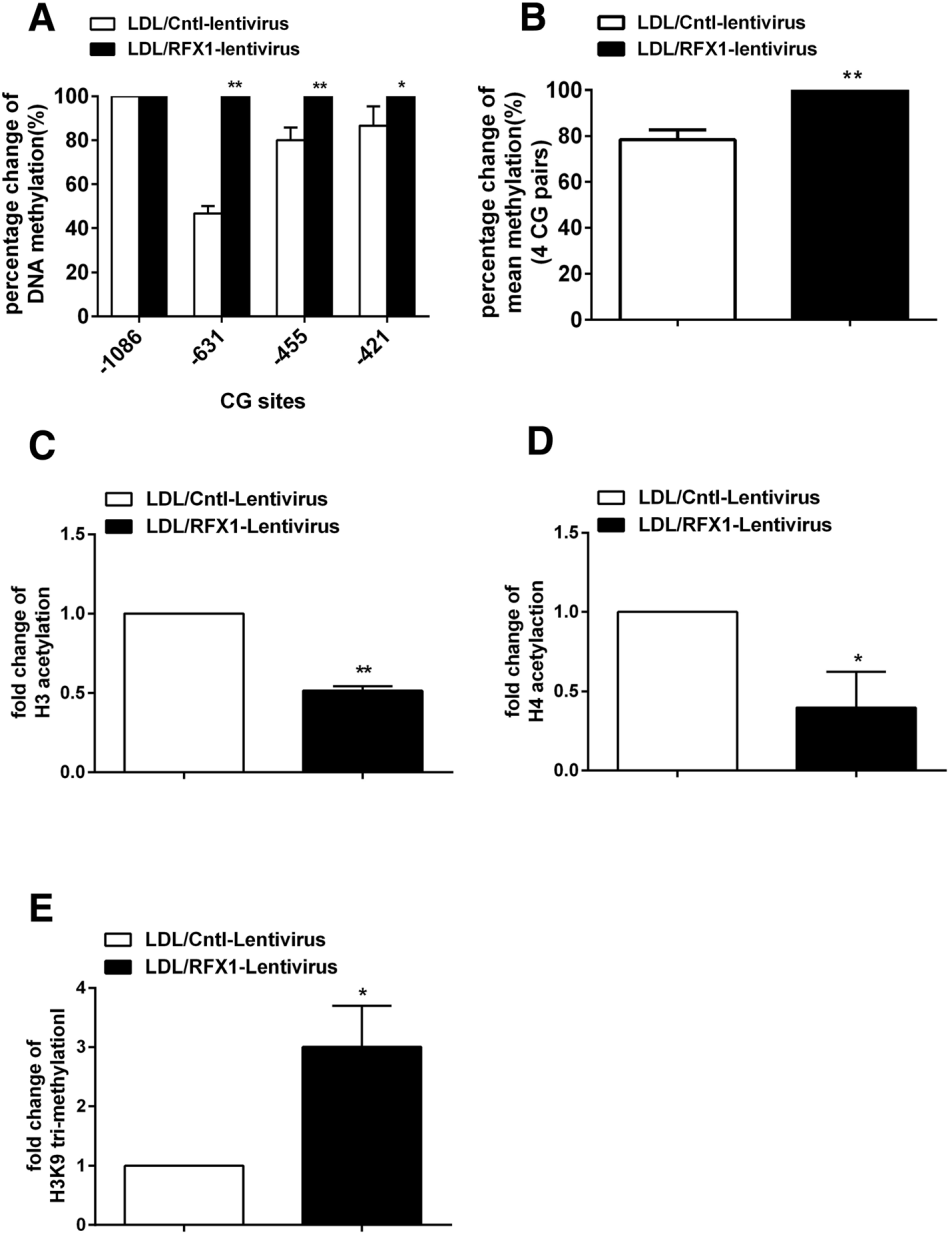
RFX1 overexpression reversed the LDL induced epigenetic modification changes of the *TLR4* promoter. **a** The percentage change of DNA methylation of four CG pairs (from − 1086 bp to − 421 bp upstream of the *TLR4* TSS) was detected by bisulfite sequencing in CD14^+^ monocytes transfected with RFX1-lentivirus compared with Cntl-lentivirus (/RFX1-lentivirus). **b** The percentage change of mean DNA methylation of all the four CG pairs (from − 1086 bp to − 421 bp upstream of the *TLR4* TSS) was detected by bisulfite sequencing in CD14^+^ monocytes transfected with RFX1-lentivirus compared with Cntl-lentivirus (/RFX1-lentivirus). **c**–**e** The fold changes of H3 (**c**), H4 (**d**) acetylation, and the H3K9 trimethylation (**e**) in *TLR4* promoter with RFX1 binding region were detected by ChIP-qPCR in CD14^+^ monocytes transfected with RFX1-lentivirus compared with Cntl-lentivirus. All values are the average of at least three biological replicates, and the data shown are the means ± SDs. *P < 0.05; **P < 0.01 relative to control

Furthermore, we detected the DNA methylation and histone modification levels of the *TLR4* promoter in the LDL-treated CD14^+^ monocytes transfected with the RFX1-expressing lentiviral vector. The bisulfite sequencing results showed that the methylation of the three CG sites − 631 bp, − 455 bp, and − 421 bp upstream of TSS of *TLR4* genes were significantly upregulated in the LDL-treated CD14^+^ monocytes transfected with RFX1-lentivirus compared with the empty control monocytes (Fig. 7a). The mean DNA methylation of the four CG sites in the *TLR4* promoter was significantly increased after RFX1-lentivirus transfection (Fig. 7b). ChIP-qPCR analysis indicated that the acetylation of H3 and H4 were significantly decreased and the trimethylation of H3K9 was significantly increased in RFX1 binding region of the promoter region of *TLR4* in the LDL-treated CD14^+^ monocytes with RFX1-lentivirus transfection compared with negative controls (Fig. 7c–e). However, no significant change was observed in the region without RFX1 binding site (Additional file 2: Figure S1D-F).

### RFX1 recruits DNMT1, HDAC1, and SUV39H1 to the *TLR4* promoter region in CD14^+^ monocytes

**Fig. 8:**
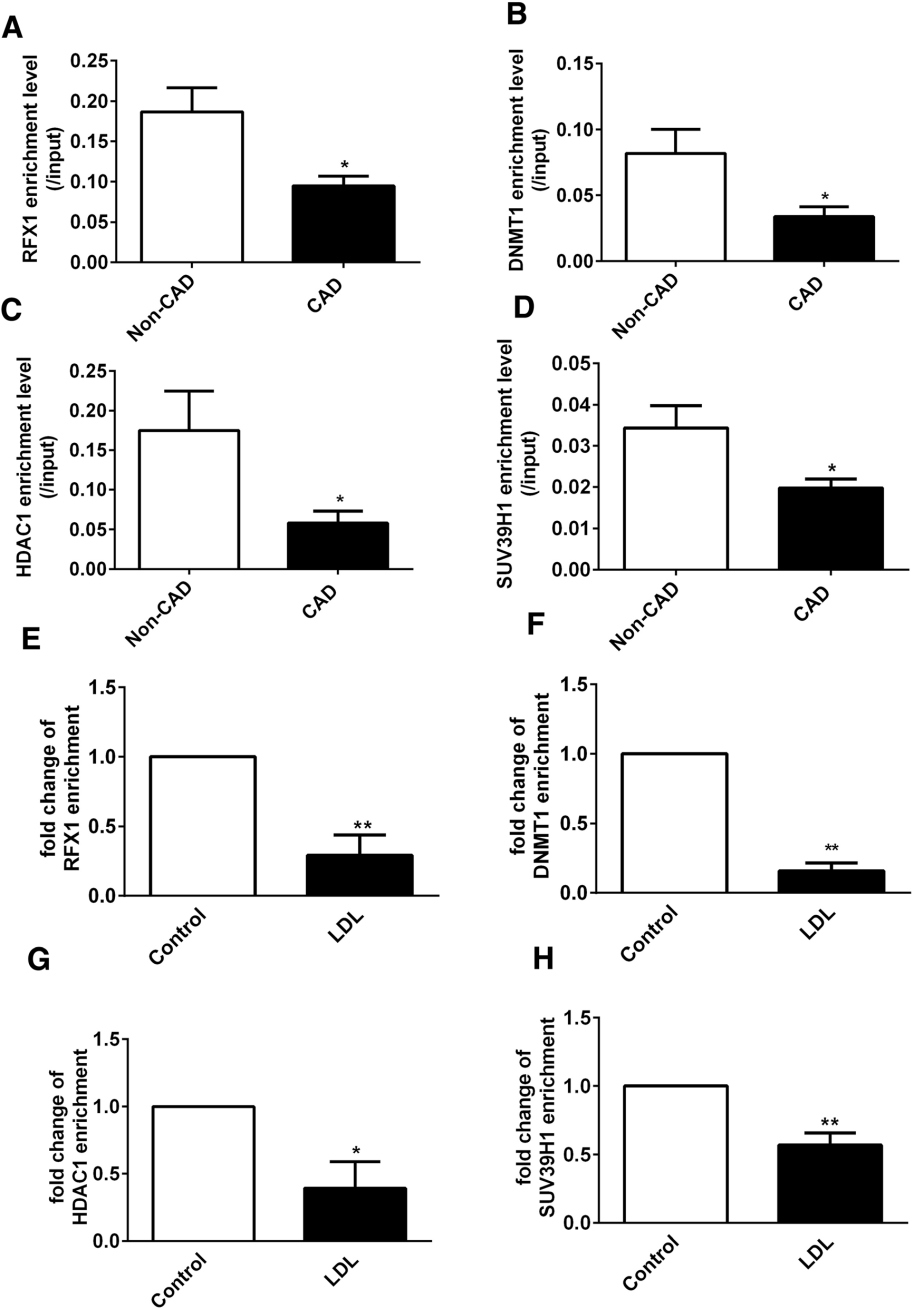
Enrichments of DNMT1, HDAC1, and SUV39H1 in the *TLR4* promoter in CD14^+^ monocytes. **a**–**d** The relative enrichment levels of RFX1 (**a**), DNMT1 (**b**), HDAC1 (**c**), and SUV39H1 (**d**) in *TLR4* promoter with RFX1 binding region were measured by ChIP-qPCR in CAD CD14^+^ monocytes. **e**–**h** The enrichments of RFX1 (**e**), DNMT1 (**f**), HDAC1 (**g**), and SUV39H1 (**h**) in *TLR4* promoter with RFX1 binding region were measured by ChIP-qPCR in the LDL-treated CD14^+^ monocytes. The fold changes were shown between LDL treatment with control group. All values are the average of at least three biological replicates, and the data shown are the means ± SDs. *P < 0.05; **P < 0.01 relative to control

**Fig. 9:**
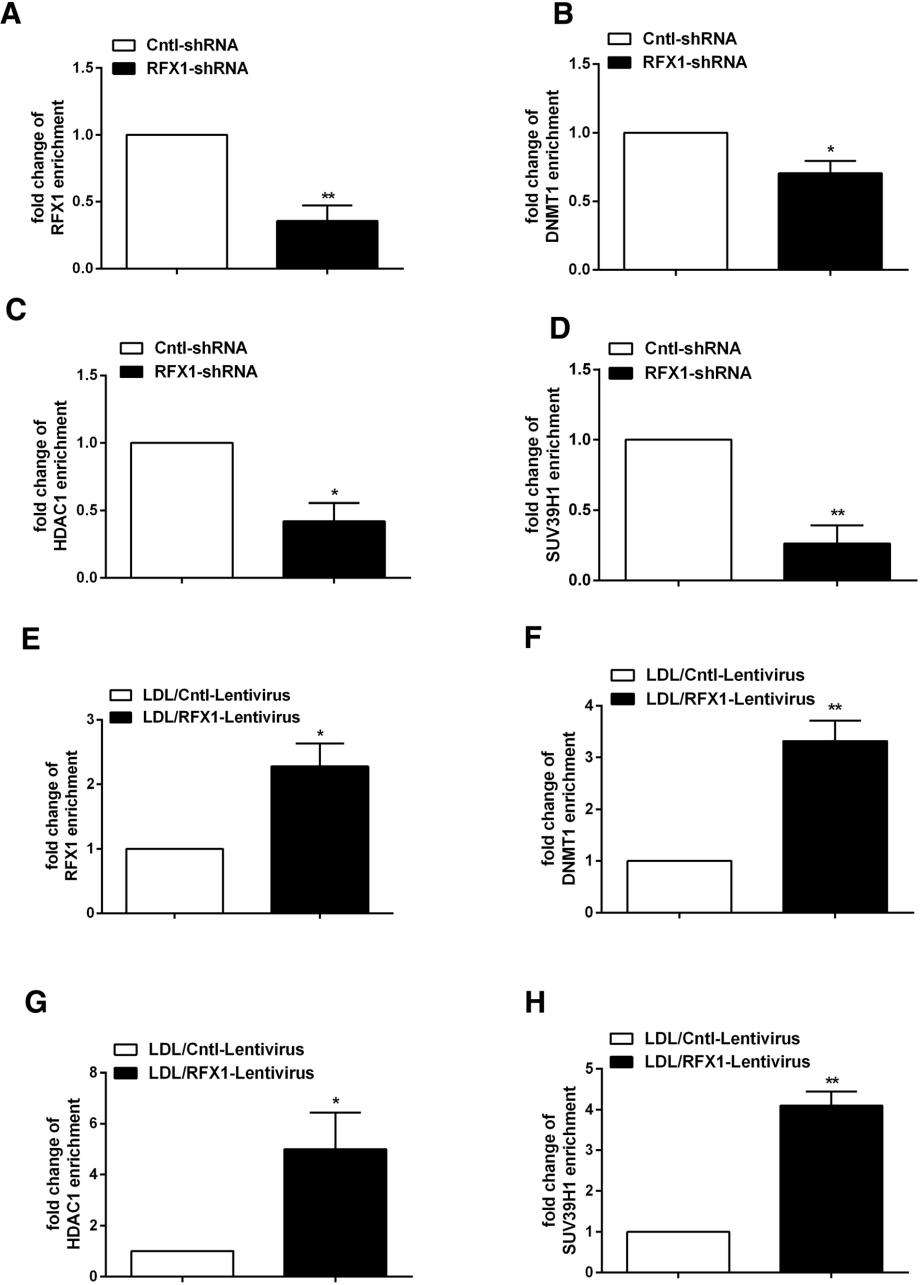
RFX1 recruits DNMT1, HDAC1, and SUV39H1 to the promoter region of *TLR4* gene. **a**–**d** The enrichments of RFX1 (**a**), DNMT1 (**b**), HDAC1 (**c**), and SUV39H1 (**d**) in *TLR4* promoter with RFX1 binding region were measured by ChIP-qPCR in CD14^+^ monocytes transfected with RFX1-shRNA or Cntl-shRNA. The fold changes were shown between RFX1-shRNA-1 group and Cntl-shRNA group. **e**–**h** The enrichments of RFX1 (**e**), DNMT1 (**f**), HDAC1 (**g**), and SUV39H1 (**h**) in *TLR4* promoter with RFX1 binding region were measured by ChIP-qPCR in CD14^+^ monocytes transfected with RFX1-lentivirus or Cntl-lentivirus. The fold changes were shown between RFX1-lentivirus group with Cntl-lentivirus group. All values are the average of at least three biological replicates, and the data shown are the means ± SDs. *P < 0.05; **P < 0.01 relative to control

To clarify the mechanism by which RFX1 regulates the aberrant epigenetic modifications of the *TLR4* promoter, we also assessed whether RFX1 recruits the epigenetic modifiers DNMT1, HDAC1, and SUV39H1 in the *TLR4* promoter region. First, we found that the enrichments of RFX1, DNMT1, HDAC1, and SUV39H1 in the *TLR4* promoter region was decreased in the CD14^+^ monocytes from the CAD patients and in the LDL-treated CD14^+^ monocytes compared with the corresponding negative controls (Fig. 8a–h). Furthermore, the ChIP-qPCR results showed that the binding of RFX1 to the *TLR4* promoter was significantly decreased in the CD14^+^ monocytes transfected with RFX1-shRNA compared with negative controls (Fig. 9a) and the enrichments of DNMT1, HDAC1, and SUV39H1 to the *TLR4* promoter in the CD14^+^ monocytes was also decreased (Fig. 9b–d). In contrast, we found that ectogenic overexpressing RFX1 increased the binding of RFX1 and the enrichments of DNMT1, HDAC1, and SUV39H1 in the LDL-treated CD14^+^ monocytes transfected with RFX1-lentivirus compared with negative controls (Fig. 9e–h). The region without RFX1 binding site has no significant change in the binding of RFX1 and the enrichments of DNMT1, HDAC1, and SUV39H1 in CD14^+^ monocytes transfected with RFX1-shRNA or RFX1-lentivirus compared with control group (Additional file 3: Figure S2).

## Discussion

In this study, we identified that RFX1 is a key regulator in the activation of CD14^+^ monocytes from CAD patients. We found that RFX1 expression was significantly decreased in CD14^+^ monocytes from CAD patients and in LDL-treated CD14^+^ monocytes. Moreover, we demonstrated that RFX1 deficiency led to the overexpression of TLR4 and the activation of CD14^+^ monocytes by inducing decreased DNA methylation and histone H3K9 trimethylation and increased H3 and H4 acetylation in the *TLR4* promoter region, which were identified in CD14^+^ monocytes of CAD patients. Moreover, our findings demonstrated that RFX1 regulates the epigenetic modifications in the *TLR4* promoter via the recruitments of the epigenetic modifiers DNMT1, HDAC1, and SUV39H1.

RFX1 is a crucial transcription factor in regulating gene expression, and has been shown to be involved in tumorigenesis, development, and immune regulation. RFX1 may be a transcriptional repressor or a transcriptional activator depending on the promoter context [27, 39–41]. RFX1 directly downregulated CD44 expression, affecting the ability for proliferation, survival, and invasion in glioblastoma cells [42]. In *Rfx1/3* conditional knockout mice, the rapid loss of initially well-formed outer hair cells induced secondary deafness, which indicates an essential role for RFX1 in hearing [43]. In systemic lupus erythematosus (SLE), RFX1 downregulated IL17A, CD70, and CD11a expression by recruiting DNMT1, HDAC1, and SUV39H1 to alter epigenetic modifications in CD4^+^ T cells from patients with lupus [28, 44]. RFX1 suppressed *IL*-*17A* gene expression, and RFX1 deficiency contributed to the increased production of IL-17 and to Th17 differentiation in SLE patient [33]. In this study, we identified a novel role of RFX1 in regulating innate immune response. We found that the expression of RFX1 was decreased in monocytes from CAD patients and that *TLR4* is a target gene of RFX1. TLR4 plays an important role in the pathogenesis of atherosclerosis [45–47]. Previous studies have demonstrated that the expression of TLR4 was markedly upregulated in human atherosclerotic plaques and peripheral blood mononuclear cells [47, 48]. TLR4 activation induced IL-6 secretion and vascular smooth muscle cell (VSMC) migration, thereby accelerating the process of AS [47]. Our results showed that the knockdown of RFX1 induced TLR4 overexpression, consequently contributing to increasing the production of related cytokines downstream of TLR4 and to monocyte activation in the pathogenesis of CAD, which suggested the vital role of RFX1 in the pathogenesis of AS and CAD.

Previous studies have indicated that epigenetic modifications regulate the overexpression of TLR4 [49]. TLR4 expression is upregulated by epigenetic events via the methylation of DNA and histones in various cells such as intestinal epithelial cells and macrophages [37, 38, 50, 51]. Moreover, previous studies have shown that aberrant epigenetic modifications were involved in AS and CAD patients. For example, the alteration of DNA methylation of different genes, such as CSE and LINE-1 [19, 20], was identified in CAD patients. The global acetylation level of histone H3 was found to increase with the severity of atherosclerosis, while the H3K9 methylation level decreased with the progression of AS [52]. Our results demonstrated that the decreased methylation of the *TLR4* promoter was involved in monocyte activation in CAD patients and LDL-treated CD14^+^ monocytes. Moreover, we found that the acetylation levels of H3 and H4 and the trimethylation level of H3K9 in the *TLR4* promoter were aberrant in CAD patients and LDL-treated CD14^+^ monocytes. Studies have demonstrated that DNA hypomethylation promoted the transcriptional activation of genes and that the acetylation of H3 and H4 facilitated the activation of gene transcription in atherosclerosis [53]. Similarly, a reduced H3K9me3 level upregulated the expression of several genes [54]. These findings indicated that TLR4 overexpression in CD14^+^ monocytes from CAD patients is due to aberrant epigenetic modifications.

The epigenetic regulation on gene expression is complex. As we all known, the epigenetic modifications include DNA methylation, histone acetylation and methylation, etc. According to a lot of previous studies, one gene expression may be regulated by these modifications simultaneously or independently. For example, Rauen et al. reported that DNA hypomethylation and the histone hyper-acetylation occur in the promoter region of *IL17A* gene in CD4^+^ T cells of SLE patients [55]. Ohm et al. found that some tumor suppressor genes expression is not only regulated by DNA methylation but also affected by histone modification during tumor initiation and progression [56]. In this study, the increased histone acetylation and the decreased DNA methylation and H3K9 trimethylation were identified in the promoter region of *TLR4* in CD14^+^ monocytes of CAD patients. Furthermore, to clarify the mechanism of the aberrantly epigenetic modifications on *TLR4* gene, we found that the transcription factor RFX1 play an important role in regulating TLR4 expression and epigenetic modifications of *TLR4* promoter. In fact, a lot of evidence shows that transcription factors mainly regulate gene expression through recruiting epigenetic modifiers such as DNMT1, HDAC1, P300, SUV39H1, etc. These epigenetic modifiers influence the status of epigenetic modifications in the target genes of transcription factors. For example, Robertson et al. reported that Rb2 can recruit DNMT1, HDAC1, and form transcription regulatory complex, thus inhibiting gene transcription [57]. Esteve et al. found that P53 recruits DNMT1 to the survivin promoter, contributing the hypermethylation of survivin promoter [58]. RFX1 is involved in the transcriptional regulation of various genes in different cells via epigenetic mechanisms [59]. Recent studies have shown that RFX1 binds to the promoter region of *IL17A*, *CD11a*, and *CD70*, altering DNA methylation, H3 acetylation, and H3K9 trimethylation to promote transcription in CD4^+^ T cells [28, 33, 44]. RFX1 recruited HDAC1 to the *COL1A2* promoter, thus repressing *COL1A2* promoter activity in IMR-90 cells [60]. In our study, we found that the knockdown of RFX1 in healthy control CD14^+^ monocytes increased histone acetylation and reduced DNA methylation and H3K9 trimethylation, which led to TLR4 overexpression. Moreover, the overexpression of RFX1 in LDL-treated CD14^+^ monocytes had the opposite effect. These findings indicated that the overexpression of TLR4 may be due to the reduction in HDAC1, DNMT1, and SUV39H1 recruitment by RFX1, which, in turn, led to an increase in histone acetylation and a decrease in DNA methylation and H3K9 trimethylation in the *TLR4* promoter region in CAD patient CD14^+^ monocytes. As the changes of three epigenetic modifications simultaneously occur in the promoter region of *TLR4* in CD14^+^ monocytes of CAD patients according to our results, all of three epigenetic modifiers DNMT1, HDAC1, and SUV39H1 were important for RFX1-mediated epigenetic regulation on TLR4 expression in CD14^+^ monocytes of CAD patients.

## Conclusions

In summary, our present study demonstrated that the decreased RFX1 expression in CD14^+^ monocytes contributed to the overexpression and the aberrant epigenetic modifications of *TLR4* genes, leading to the activation of monocytes in CAD patients. These novel findings highlighted the important role of RFX1 in regulating the innate immune response in CAD and provided a potential target for CAD therapy.

## Methods

### Subjects

Patients who were diagnosed with CAD by angiography (at least one coronary artery with a stenosis ≥ 50% of the diameter) and age- and sex-matched angiographically defined non CAD patients (without coronary artery with a stenosis ≥ 50% of the diameter) were recruited at the Third Xiangya Hospital. The healthy volunteers were recruited from the medical staffs at the Third Xiangya Hospital. The characteristics of the patients are shown in Table 1. The exclusion criteria were as follows: participants with cardiomyopathy, using of statins before blood sampling, peripheral vascular diseases, autoimmune disease, thyroid diseases, severe kidney or liver disease, or malignant disease. This study was approved by the Ethics Committee of the Third Xiangya Hospital of Central South University, and written informed consent was obtained from all subjects.

**Table 1:** Clinical characters of CAD patients and non-CAD subjects

Characteristics	CAD (*n* = 26)	Non-CAD (*n* = 24)
	(Mean ± SD)	(Mean ± SD)
Age (years)	61.31 ± 8.94	62.04 ± 7.95
Gender (M/F)	(11/15)	(14/10)
Hypertension, *n* (%)	16 (61.5)	13 (54.2)
Diabetes, *n* (%)	9 (34.6)	4 (16.7)
SBP (mmHg)	134.83 ± 18.24	132.75 ± 11.80
DBP (mmHg)	80.92 ± 9.95	82.28 ± 9.43
HR (bpm)	80.50 ± 6.45	72.28 ± 7.33
BNP (pg/mL)	920.04 ± 2256.91	958.80 ± 2348.33
MONO (10^9^/L)	0.48 ± 0.21	0.39 ± 0.14
TC (mmol/l)	4.75 ± 0.83	4.44 ± 0.94
TG (mmol/l)	1.94 ± 1.38	1.47 ± 0.57
HDL-C (mmol/l)	1.22 ± 0.27	1.15 ± 0.27
LDL-C (mmol/l)	2.59 ± 0.58	2.45 ± 0.67
FBS (mmol/l)	5.47 ± 1.38	5.31 ± 0.95
ALT(U/L)	33.13 ± 28.81	28.29 ± 15.83
AST(U/L)	43.35 ± 45.35	23.42 ± 7.30
TBIL (μmol/l)	13.71 ± 5.63	14.94 ± 5.89
CB (μmol/l)	4.33 ± 2.44	3.99 ± 1.93
TP (g/L)	65.77 ± 4.27	64.32 ± 5.78
ALB (g/L)	40.02 ± 2.78	40.26 ± 4.15
GLO (g/L)	27.46 ± 9.22	24.06 ± 4.02
A/G	3.26 ± 7.42	1.73 ± 0.34
UREA (mmol/l)	4.62 ± 1.53	4.90 ± 1.34
CRE (μmol/l)	73.79 ± 16.53	80.92 ± 17.89
UA (μmol/l)	329.71 ± 90.43	308.42 ± 85.88

*SBP* indicates systolic blood pressure, *DBP* diastolic blood pressure, *HR* heart rate, *BNP* brain natriuretic peptide, *MONO* monocyte count, *TC* total cholesterol, *TG* triglyceride, *HDL*-*C* high-density lipoprotein cholesterol, *LDL*-*C* low-density lipoprotein cholesterol, *FBG* Fasting blood glucose, *ALT* alanine aminotransferase, *AST* Aspartate aminotransferase, *TBIL* total bilirubin, *CB* conjugated bilirubin, *TP* total protein, *ALB* albumin, *GLO* globulin, *A*/*G* albumin/globulin, *UREA* serum urea, *CRE* creatinine, *UA* serum uric acid

### Isolation of CD14^+^ monocyte cells

Peripheral blood mononuclear cells (PBMCs) were isolated via Ficoll-Hypaque density gradient centrifugation (GE Healthcare, Boston, MA, USA). CD14^+^ monocytes were then isolated from PBMCs by positive selection using magnetic beads (Miltenyi Biotec, Bergisch Gladbach, Germany; the purity was generally greater than 95%) according to the protocol provided by the manufacturer.

### Cell culture

All CD14^+^ monocytes and THP-1 cells were cultured in RPMI 1640 medium (Gibco, New York, USA) supplemented with 10% fetal bovine serum (FBS, Capricorn Scientific, USA). Human umbilical vein endothelial cells (HUVECs) were cultured with DMEM basic (1×) (Gibco, New York, USA) that contained 10% fetal bovine serum. The cultures were incubated at 37 °C in a humidified atmosphere, which contained 5% CO_2_.

### Monocyte treatment and transfection

The CD14^+^ monocytes were seeded at a density of 1 × 10^6^ cells/mL in a 12-well plate, and LDL (Yiyuan Biotechnologies, Guangzhou, China) was added at a final concentration of 100 mg/L. CD14^+^ monocytes were transfected with lentivirus (Genechem, Shanghai, China). The in vitro cell culture experiments were composed of three parts. The first part detected the effect of LDL treatment on RFX1 expression in CD14^+^ monocytes. Monocytes were randomly assigned to two groups: the negative control group (treated with PBS) and the LDL-treated group (treated with LDL for 24 h). The second part detected the effect of RFX1 overexpression on LDL-treated monocytes. Monocytes were randomly assigned to two groups; both groups of CD14^+^ monocytes were initially cultured with LDL at 100 mg/L, and after 24 h, RFX1-expressing lentivirus (LDL/RFX1-lentivirus group) or empty lentivirus (LDL/Cntl-lentivirus group) was added to the monocytes by gentle suspension following the determination of the multiplicity of infection (MOI = 5). The third part detected the effect of RFX1 knockdown on CD14^+^ monocytes. Prior to adding RFX1-shRNA, CD14^+^ monocytes were harvested and seeded at a density of 3 × 10^6^ cells/mL in a 6-well plate. Monocytes were transfected with negative control or RFX1-shRNA at the appropriate MOI (MOI = 5). Transfected cells were cultured in human monocyte cell culture medium and harvested after 72 h.

### RNA isolation and real-time quantitative polymerase chain reaction

Total RNA was isolated from CD14^+^ monocytes using TRIzol reagent (Invitrogen, Carlsbad, CA, USA) according to the manufacturer’s instructions. Real-time quantitative polymerase chain reaction (RT-qPCR) was performed using a LightCycler 96 (Roche, Basel, Switzerland), and the mRNA levels were quantified using a SYBR Prime Script RT-qPCR kit (Takara, Dalian, China). β-actin was also amplified and used as a loading control. The relative expression of mRNA was calculated using 2^-ΔCt^ (ΔCt = Ct_target gene_ - Ct_β-actin_) method in CAD patients compared with non-CAD patients and the fold change of mRNA expression level was calculated using 2^-ΔΔCt^ method (ΔΔCt = ∆Ct_experiment group_ - ∆Ct_control group_) in experiment group compared with control group. All data was normalized to internal control β-actin. The sequences of the primers are shown in Additional file 1: Table S1.

### Western blotting

CD14^+^ monocytes were lysed with whole cell lysis buffer and denatured at 100 °C for 5 min. Protein was then separated by SDS polyacrylamide gel electrophoresis and transferred onto PVDF membranes. The membranes were blocked in PBST buffer that contained 5% nonfat dry milk and were incubated overnight at 4 °C with an anti-TLR4 antibody (1:1000, Abcam, Cambridge, UK), anti-RFX1 antibody (1:1000, Genetex, Irvine, CA, USA), or anti-β-actin antibody (1:1000, Santa Cruz, Dallas, Texas, USA). The experiments were repeated three times, and the relative protein expression level was quantified with band density value normalized to β-actin.

### Genomic DNA extraction and genomic bisulfite sequencing

Genomic DNA extraction was carried out with a TIANamp Genomic DNA blood kit (Tiangen Biotech, Beijing, China). Genomic DNA was subjected to sodium bisulfite modification using an EpiTect bisulfite kit (Qiagen, Valencia, CA, USA), and nested PCR was carried out with a GoTaq hot start polymerase system (Promega, Madison, WI, USA). The sequences of the primers are shown in Additional file 1: Table S1. The outer primer pairs were 1013 bp; the inner primer pairs were 587 bp, with four CpGs in the products. The PCR products were purified by gel electrophoresis and subcloned into a pGEM®-T Easy Vector System (Promega, Madison, WI, USA) for Sanger sequencing by an ABI 3730.

### Chromatin immunoprecipitation assays

ChIP analysis was performed according to the instructions provided with the ChIP assay kit (Millipore, Billerica, MA, USA). In brief, CD14^+^ monocytes were fixed for 10 min at RT with 1% formaldehyde. Glycine was subsequently added to a final concentration of 0.125 M to quench the formaldehyde. Cells were pelleted, washed once with ice-cold PBS, and lysed with SDS buffer. Lysates were pelleted, resuspended, and sonicated to reduce DNA to fragments of 200 to 1000 base pairs. Chromatin was precipitated with protein A agarose beads for 1 h and then incubated with an anti-RFX1 antibody (Santa Cruz), anti-DNMT1 antibody (Abcam), anti-HDAC1 antibody (Abcam), anti-SUV39H1 antibody (Abcam), histone H3ac (pan-acetyl) antibody (Active motif), histone H4ac (pan-acetyl) antibody (Active motif), and histone H3 trimethyl Lys9 antibody (Active motif) or normal IgG (Millipore, Darmstadt, Germany) overnight. The immunocomplexes were further precipitated with protein A agarose beads, washed, and eluted in 100 mL of TE with 0.5% SDS and 200 mg/mL proteinase K. Precipitated DNA was further purified with phenol/chloroform extraction and ethanol. The relative enrichment level was quantified using qPCR and calculated relative to the respective input DNA [33]. The primers used are shown in Additional file 1: Table S1.

### Monocyte–endothelial adhesion assays

Human umbilical vein endothelial cells (HUVECs) were seeded into 12-well plates at a density of 3 × 10^5^ cells/mL for 1 day prior to assays, and the confluence was confirmed before the adhesion studies. CD14^+^ monocytes (5 × 10^5^ cells/mL) stained with 5 μM BCECF-AM (Beyotime, Jiangsu, China) in the dark at 37 °C for 30 min were added onto confluent monolayers of the HUVECs and incubated for 30 min. After nonadherent cells were removed, the plates were washed with PBS twice. The cell numbers were subsequently counted in five random fields per well at × 200 magnification using a Leica CTR 4000 microscope and LAS V4.5 software. The average number of adherent monocytes was calculated using the software Image-Pro Plus 6.0 (Media Cybernetics, Rockville, MD, USA).

### Transwell assays

The migration assay was performed using a Transwell insert (Corning Incorporated, New York, USA; pore size, 8 μm) in 24-well plates. MCP-1 was dissolved in PBS that contained 0.1% bovine serum albumin (BSA). CD14^+^ monocytes (5 × 10^5^ cells in 200 μL RPMI 1640 medium) were placed in the upper chamber, and RPMI 1640 medium supplemented with 5% FBS that contained MCP-1 at a final concentration of 200 ng/mL was placed in the lower chamber. The plates were incubated for 90 min at 37 °C in 5% CO_2_, after which the culture medium was removed and the filters were washed with PBS twice. The cells on the upper side of the filters were removed using cotton-tipped swabs, and the cells on the underside of the filters were fixed in 95% alcohol for 10 min and stained with crystal violet staining solution (Beyotime, Jiangsu, China) for 10 min at room temperature. The cells that had migrated from the upper to the lower side of the filter were counted in five random fields of view for each well at × 100 magnification under a Leica CTR 4000 microscope with LAS V4.5 software. The monocyte cell migration assay was performed in triplicate.

### Luciferase reporter assays

To generate the human *TLR4* promoter luciferase reporter construct, the proximal 1000 bp DNA fragment upstream of the transcription start site (TSS) with the RFX1 binding site and the DNA fragments (from − 1000 bp upstream of the TSS) with a mutant RFX1 binding site (from − 99 bp to − 95 bp upstream of the TSS) were directly synthesized and cloned into the pGL3-Basic vector upstream of the luciferase reporter gene (TLR4-luc WT or MU). The day before transfection, 5 × 10^4^ HEK293T cells (ATCC, CRL-11268™) were seeded into 24-well culture plates in fresh medium. Cells were separately cotransfected with 500 ng of two types of recombinant plasmids (TLR4-luc WT or MU) together with empty vectors (blank control) or plasmid-encoded RFX1 cDNA. Each reporter experiment included 100 ng of renilla luciferase construct as an internal control. Forty-eight hours after cotransfection, the cells were lysed and assayed using a Dual-Luciferase® Reporter Assay kit (Promega, Madison, WI, USA) based on the manufacturer’s protocols.

### Statistical analysis

The data were expressed as the mean ± standard deviation (SD). Variables were compared using Student’s *t* tests (data from different transfections were compared by a paired *t* test, and other data were compared by an independent samples *t* test). For a comparison of ≥ 3 groups, one-way ANOVA was used. Correlations were determined using Pearson’s correlation coefficient. All analyses were performed with GraphPad Prism 6.0 and SPSS 19.0 software (SPSS, Inc., Chicago, IL). *P* < 0.05 was considered significant.

## Additional files

Additional file 1: **Table S1.** Primer sequences. (DOCX 14 kb)

Additional file 2: **Figure S1.** The changes of H3 acetylation, H4 acetylation and H3K9 trimethylation in the distal promoter of *TLR4* gene without RFX1 binding site. A-D, The enrichments of H3 acetylation (A), H4 acetylation (B) and H3K9 trimethylation (C) in the region without RFX1 binding site were measured by ChIP-qPCR in CD14^+^ monocytes transfected with RFX1-shRNA-1 or Cntl-shRNA. The fold changes were shown between RFX1-shRNA-1 group and Cntl-shRNA group. D-F, The enrichments of H3 acetylation (D), H4 acetylation (E) and H3K9 trimethylation (F) in the region without RFX1 binding site were measured by ChIP-qPCR in LDL-treated CD14^+^ monocytes transfected with RFX1-lentivirus or Cntl-lentivirus. The fold changes were shown between RFX1-lentivirus group with Cntl-lentivirus group. All values are the average of at least 3 biological replicates, and the data shown are the means ± SDs. * *P* < 0.05, ** *P* < 0.01 relative to control. (JPG 1056 kb)

Additional file 3: **Figure S2.** The enrichments of RFX1, DNMT1, HDAC1, and SUV39H1 in the distal promoter of *TLR4* gene without RFX1 binding site. A-D, The enrichments of RFX1 (A), DNMT1 (B), HDAC1 (C) and SUV39H1 (D) in the region without RFX1 binding site were measured by ChIP-qPCR in CD14^+^ monocytes transfected with RFX1-shRNA or Cntl-shRNA. The fold changes were shown between RFX1-shRNA-1 group and Cntl-shRNA group. E-H, The enrichments of RFX1 (E), DNMT1 (F), HDAC1 (G) and SUV39H1 (H) in the region without RFX1 binding site were measured by ChIP-qPCR in LDL-treated CD14^+^ monocytes transfected with RFX1-lentivirus or with Cntl-lentivirus. All values are the average of at least 3 biological replicates, and the data shown are the means ± SDs. * *P* < 0.05, ** *P* < 0.01 relative to control. (JPG 1769 kb)

## Abbreviations

ABCA1: ATP-binding cassette A1
ACS: Acute coronary syndrome
AS: Atherosclerosis
CAD: Coronary artery disease
CSE: Cystathionine-gamma-lyase
CVD: Cardiovascular disease
DNA: Deoxyribonucleic acid
DNMT1: DNA methyltransferase 1
HDAC1: Histone deacetylase 1
LDL: Low-density lipoprotein
LINE-1: Long interspersed element-1
ox-LDL: Oxidized low density lipoprotein
PBMCs: Peripheral blood mononuclear cells
RFX1: Regulatory factor X1
SLE: Systemic lupus erythematosus
SUV39H1: Histone-lysine *N*-methyltransferase SUV39H1
TLR4: Toll-like receptor 4
TSS: Transcription start site
