# Supplementary material for: RFX1 downregulation contributes to TLR4 overexpression in CD14+ monocytes via epigenetic mechanisms in coronary artery disease
Source: Clin Epigenetics. 2019 Mar 11;11:44. doi: 10.1186/s13148-019-0646-9 (PMC6413463; doi:10.1186/s13148-019-0646-9)
Supplement: Supplementary file 1 — Table S1. Primer sequences. (DOCX 14 kb) [file 13148_2019_646_MOESM1_ESM.docx]

**Additional file 1**

**Table S1. Primer sequences**

| Gene | Primer | Sequence (5’–3’) | Application |
| --- | --- | --- | --- |
| TLR4 | Forward | TACAAAATCCCCGACAACCTCC | RT-qPCR |
|  | Reverse | GCTGCCTAAATGCCTCAGGG |  |
| TNF-α | Forward | CTCCTTCAGACACCCTCAACCT | RT-qPCR |
|  | Reverse | CGACCCTAAGCCCCCAATT |  |
| IL-6 | Forward | ATTCGGTACATCCTCGACGGC | RT-qPCR |
|  | Reverse | GCCAGTGCCTCTTTGCTGCTTT |  |
| MCP-1 | Forward | CATCTCCTACACCCCACGAA | RT-qPCR |
|  | Reverse | GGGTTGGCACAGAAACGTC |  |
| β-actin | Forward | GCACCACACCTTCTACAATGAGC | RT-qPCR |
|  | Reverse | GGATAGCACAGCCTGGATAGCAAC |  |
| TLR4 | Forward | ATATTATTTGTGGGAATGTAAAATGGATA | BSP (outer pairs) |
|  | Reverse | TTAAAACCCACCATAATAACCTCAT |  |
| TLR4 | Forward | TTAAAAGAATTGAAAATAGAGATTTTAAGA | BSP (inner pairs) |
|  | Reverse | AACTCTCTCTCCATATACAATCATAATCTA |  |
| TLR4 | Forward | GGTCATTATGGTGGGCCCTA | ChIP-qPCR  (RFX1 binding region) |
|  | Reverse | AGGTGTCGCAAGTGTGTCT |  |
| TLR4 | Forward | AACGGTGTCTACTTGGGTGT | ChIP-qPCR  (without RFX1 binding site) |
|  | Reverse | TGGCATCTGGGAAGAGACTG |  |
