# Supplementary figures and images for: RFX1 downregulation contributes to TLR4 overexpression in CD14+ monocytes via epigenetic mechanisms in coronary artery disease
Source: Clin Epigenetics. 2019 Mar 11;11:44. doi: 10.1186/s13148-019-0646-9 (PMC6413463; doi:10.1186/s13148-019-0646-9)

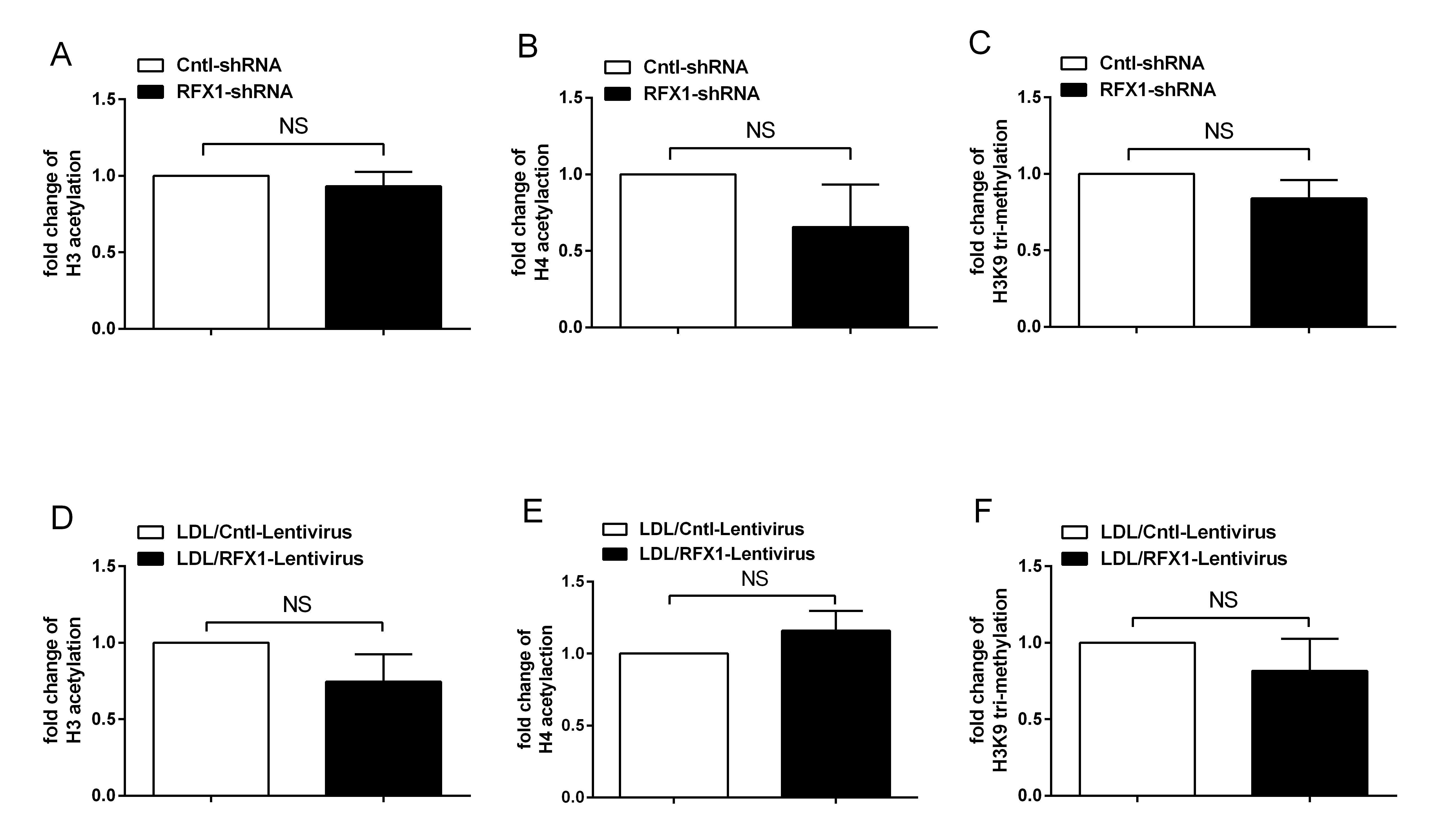

Supplement: Supplementary file 2 — Figure S1. The changes of H3 acetylation, H4 acetylation and H3K9 trimethylation in the distal promoter of TLR4 gene without RFX1 binding site. A-D, The enrichments of H3 acetylation (A), H4 acetylation (B) and H3K9 trimethylation (C) in the region without RFX1 binding site were measured by ChIP-qPCR in CD14+ monocytes transfected with RFX1-shRNA-1 or Cntl-shRNA. The fold changes were shown between RFX1-shRNA-1 group and Cntl-shRNA group. D-F, The enrichments of H3 acetylation (D), H4 acetylation (E) and H3K9 trimethylation (F) in the region without RFX1 binding site were measured by ChIP-qPCR in LDL-treated CD14+ monocytes transfected with RFX1-lentivirus or Cntl-lentivirus. The fold changes were shown between RFX1-lentivirus group with Cntl-lentivirus group. All values are the average of at least 3 biological replicates, and the data shown are the means ± SDs. * P < 0.05, ** P < 0.01 relative to control. (JPG 1056 kb) [file 13148_2019_646_MOESM2_ESM.jpg]

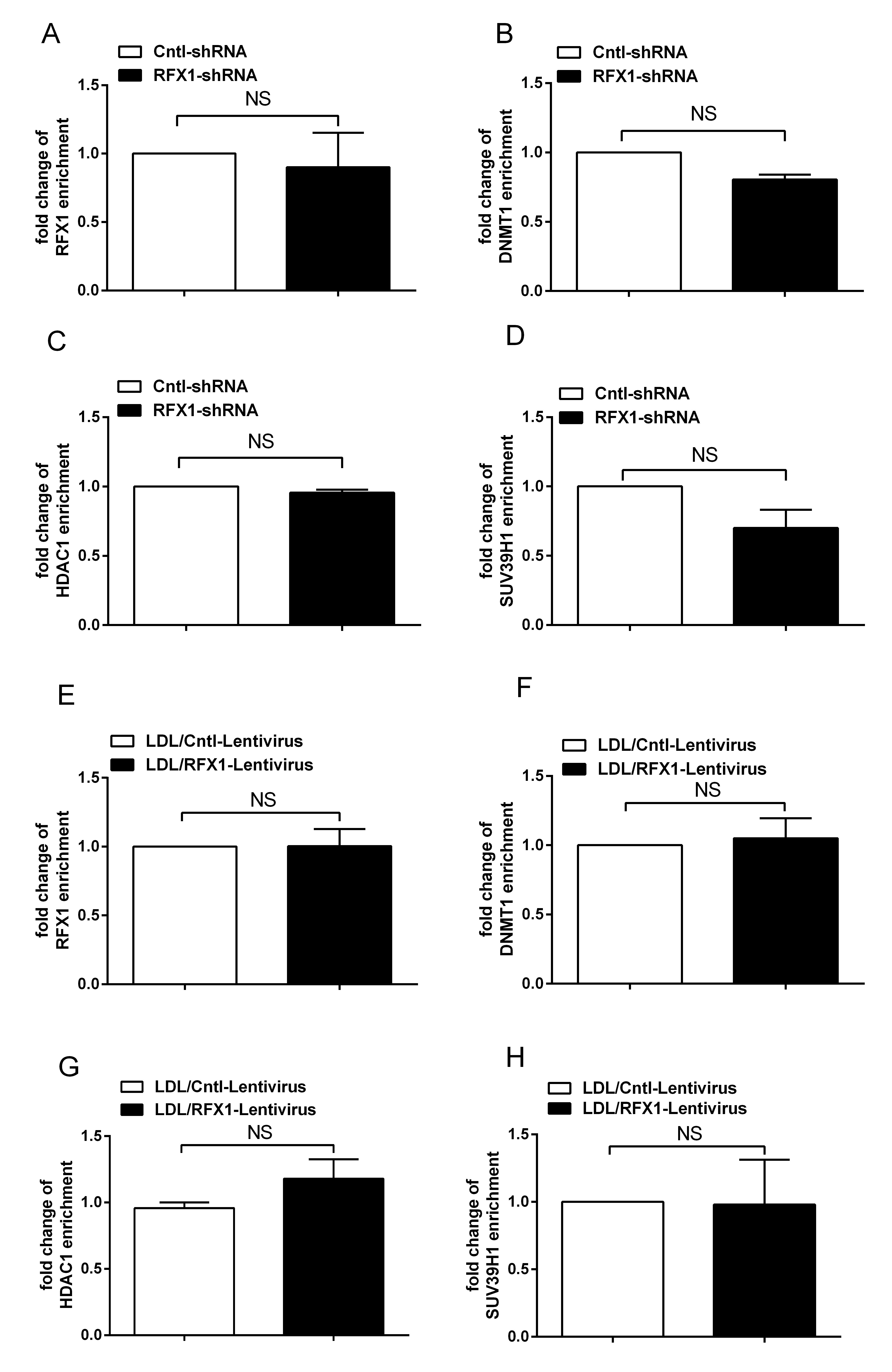

Supplement: Supplementary file 3 — Figure S2. The enrichments of RFX1, DNMT1, HDAC1, and SUV39H1 in the distal promoter of TLR4 gene without RFX1 binding site. A-D, The enrichments of RFX1 (A), DNMT1 (B), HDAC1 (C) and SUV39H1 (D) in the region without RFX1 binding site were measured by ChIP-qPCR in CD14+ monocytes transfected with RFX1-shRNA or Cntl-shRNA. The fold changes were shown between RFX1-shRNA-1 group and Cntl-shRNA group. E-H, The enrichments of RFX1 (E), DNMT1 (F), HDAC1 (G) and SUV39H1 (H) in the region without RFX1 binding site were measured by ChIP-qPCR in LDL-treated CD14+ monocytes transfected with RFX1-lentivirus or with Cntl-lentivirus. All values are the average of at least 3 biological replicates, and the data shown are the means ± SDs. * P < 0.05, ** P < 0.01 relative to control. (JPG 1769 kb) [file 13148_2019_646_MOESM3_ESM.jpg]
